# Ultrasonic spectrum analysis for in vivo characterization of tumor microstructural changes in the evaluation of tumor response to chemotherapy using diagnostic ultrasound

**DOI:** 10.1186/1471-2407-13-302

**Published:** 2013-06-21

**Authors:** Chun-yi Lin, Long-hui Cao, Jian-wei Wang, Wei Zheng, Yao Chen, Zi-zhen Feng, An-hua Li, Jian-hua Zhou

**Affiliations:** 1 Department of Ultrasound, State Key Laboratory of Oncology in South China, Sun Yat-Sen University Cancer Center, Guangzhou, 510060, P.R. China; 2 School of Electronic and Information Engineering, South China University of Technology, Guangzhou, 510640, P.R. China; 3 Department of Anesthesiology, State Key Laboratory of Oncology in South China, Sun Yat-Sen University Cancer Center, Guangzhou, 510060, P.R. China; 4 Department of Radiation Oncology, State Key Laboratory of Oncology in South China, Sun Yat-Sen University Cancer Center, Guangzhou, 510060, P.R. China

**Keywords:** Adriamycin, Chemotherapy, Cancer, Ultrasonic spectrum analysis, Microstructure

## Abstract

**Background:**

There is a strong need for early assessment of tumor response to chemotherapy in order to avoid the adverse effects of unnecessary chemotherapy and to allow early transition to second-line therapy. The purpose of this study was to determine the feasibility of ultrasonic spectral analysis for the in vivo characterization of changes in tumor microstructure in the evaluation of tumor response to chemotherapy using diagnostic ultrasound.

**Methods:**

Experiments were approved by the regional animal care committee. Twenty-four MCF-7 breast cancer bearing nude mice were treated with adriamycin or sterile saline administered by intraperitoneal injection. Ultrasonic radio-frequency (RF) data was collected using a clinically available ultrasound scanner (6-MHz linear transducer). Linear regression parameters (spectral slope and midband-fit) regarding the calibrated power spectra from the RF signals were tested to monitor tumor response to treatment. The section equivalent to the ultrasound imaging plane was stained with hematoxylin and eosin to allow for assessment of the density of tumor cell nuclei.

**Results:**

Treatment with adriamycin significantly reduced tumor growth in comparison with the control group (*p* = 0.003). Significant changes were observed in the ultrasonic parameters of the treated relative to the untreated tumors (*p* < 0.05). The spectral slope increased by 48.5%, from −10.66 ± 2.96 to −5.49 ± 2.69; the midband-fit increased by 12.8%, from −57.10 ± 7.68 to −49.81 ± 5.40. Treated tumors were associated with a significant decrease in the density of tumor cell nuclei as compared with control tumors (*p <* 0.001).

**Conclusions:**

Ultrasonic spectral analysis can detect changes in tumor microstructure after chemotherapy, and this will be helpful in the early evaluation tumor response to chemotherapy.

## Background

Tumor malignancy is one of the principal diseases that adversely affect human health and quality of life. More than half of all patients diagnosed with a malignant tumor will receive chemotherapy. At the present time, chemotherapy is still one of the most important cancer treatment methods. Early evaluation of tumor response to chemotherapy in patients with cancer may help to avoid unnecessary treatment and enable the use of alternative therapies. Currently in clinical oncology and experimental therapeutics, assessment of tumor treatment response to chemotherapy relies on evaluating changes in tumor growth rate or volume weeks to months after the conclusion of a therapeutic protocol. These changes typically occur weeks to months late in the course of therapy. Functional techniques such as positron-emission tomography (PET), dynamic contrast-enhanced magnetic resonance imaging (MRI) and dynamic contrast-enhanced computed tomography (CT) have been investigated regarding the early assessment of tumor response to chemotherapy; this involves depiction of reductions in the metabolic activity or the perfusion of the tumor, and some of the results have been promising [1-3]. However, the use of such imaging modalities to monitor tumor responses to chemotherapy can be limited either by their cost or exposure of the patient to radiation.

The aim of cancer therapy is to kill tumors by inducing cell death that can be used as an indicator of tumor response to therapy [4]. Increased tumor cell death early during the course of treatment, in preclinical and clinical studies, has been shown to be a good prognostic indicator of outcome [5,6]. Currently, standard methods for detecting cell death are invasive and require tissue biopsy for histologic analysis. Diffusion-weighted MRI has been used clinically to measure the increase in the apparent diffusion coefficient of water, which is thought to be increased in responding tumors owing to a decrease in cell density [7,8]. However, assessment of tumor response to chemotherapy requires repeated examinations and the cost of MRI limits its clinical use.

Ultrasound is an attractive modality for the assessment of tumor response to therapy because of the ease with which it can be repeated without exposing the patient or animal to any risk of radiation. Ultrasound imaging systems are also relatively inexpensive and mobile, a particular benefit for animal studies. Ultrasonic spectrum analysis is used to extract information regarding tissue structures that is not conveyed in conventional B-mode imaging. Spectrum analysis of frequency-dependent backscattered radiofrequency (RF) data has been used to characterize tissue microstructures in the diagnosis of prostate cancer, ocular tumors, and cardiac abnormalities [9-11] and to differentiate benign lymph nodes from malignant lymph nodes [12]. It has been shown theoretically that the spectral parameters are related to tissue microstructural properties (e.g., effective acoustic scatterer size and concentration) [13]. Ultrasonic spectral analysis with a high frequency transducer has been used to detect microstructural changes induced by radiotherapy, photodynamic therapy and chemotherapy in tumor xenografts [14-17]. Recently, Sadeghi-Naini and colleagues used ultrasonic spectral analysis with a Sonix RP system at an approximate center frequency of 7 MHz to examine response to neoadjuvant chemotherapeutic in breast cancer patients and the preliminary results were promising [18]. However, this study was limited by the small number of cases (n = 10) and no confirmation of the changes in spectral parameters was obtained from evaluation of the corresponding tissue microstructures. The purpose of the present study was to determine the feasibility of ultrasonic spectral analysis for the in vivo characterization of tumor microstructural changes in the evaluation of tumor response to chemotherapy using diagnostic ultrasound.

## Methods

### Animal model

This study was approved by the Committee on the Ethics of Animal Experiments of the Sun Yat-Sen University under the guidelines of the National Institutes of Health for the care of laboratory animals. Human breast cancer cell line MCF-7 was obtained from State Key Laboratory of Oncology in Southern China. MCF-7 cells were grown in DMEN culture medium (Hyclone Co., UT, USA) supplemented with 10% fetal bovine serum (Gibco, Grand Island, NY, USA), penicillin (50 U/ml), and streptomycin (50 μg/ml) at 37°C in a humidified 5% CO_2_ atmosphere. For inoculation, approximately 4 × 10^8^ MCF-7 cells suspended in phosphate-buffered saline were injected subcutaneously into the right axillary fossa of 24 5- to 6-week old BALB/c nude female mice.

Twenty-four nude mice were randomly divided into two groups. Twelve of the nude mice were treated with adriamycin (Shenzhen Main Luck Pharmaceuticals Inc., Guangdong, China) diluted in sterile saline and administered once daily by intraperitoneal injection (4 mg/kg). Drug administration began at day 10 post tumor cell implantation when tumors had reached a size of 5 mm. The remaining twelve nude mice in the control group were given the vehicle control medium (sterile saline) according the timing and dosing schedule used for the treated group.

### Ultrasound data acquisition

Ultrasound imaging was performed at day 18 (i.e. 7 days after initiation of therapy). For the ultrasound imaging studies, each mouse was anesthetized by intraperitoneal injection of pentobarbital sodium at a dose of 75 mg/kg (Sigma, St. Louis, MO, USA). Centrifuged gel was used to minimize bubble formation in the gel and a stand-off gel pad was placed on the skin for scanning. A commercially available clinical ultrasound scanner, Sonix TOUCH (Ultrasonix Medical Corporation, Richmond, Canada) with a 6-MHz linear transducer was used to simultaneously collect B-mode images and RF data from the treated and control tumors. For data acquisition, the ultrasound transducer was positioned such that the focal zone was at the same depth in each imaged specimen to control for any potential attenuation. All RF data were sampled with 16 bit resolution at a frequency of 35 MHz. All images and radiofrequency data were digitally recorded. All ultrasound examinations were performed by one radiologist, who was blinded to the treatment status. The greatest longitudinal, transverse and anteroposterior dimensions of tumors were measured in fundamental grayscale imaging using calipers. Tumor volume was calculated using the formula for a prolate ellipsoid: volume = π/6 × length × width × depth. The largest cross-section plane of the tumor was imaged with the transducer held manually in this position throughout the examination. To evaluate the echogenicity changes in the tumor after chemotherapy, Adobe Photoshop 6.0 (Adobe Systems, San Jose, CA, USA) was used to measure the gray scale intensity of the ultrasound images.

### Ultrasonic spectral analysis

The ultrasound RF data from was imported into MATLAB-based (v. 2009a: MathWorks, Natick, MA, USA) software developed in our lab for ultrasound spectral analysis. For the tumor, rectangular regions of interest (ROI) were centered approximately at the focal depth of the transducer. Three representative ROIs were selected for each tumor sample and averaged for the final analysis. RF data from each line segment were multiplied by a Hamming weighting function to suppress spectral lobes and the Fourier transform was computed.

**Figure 1:**
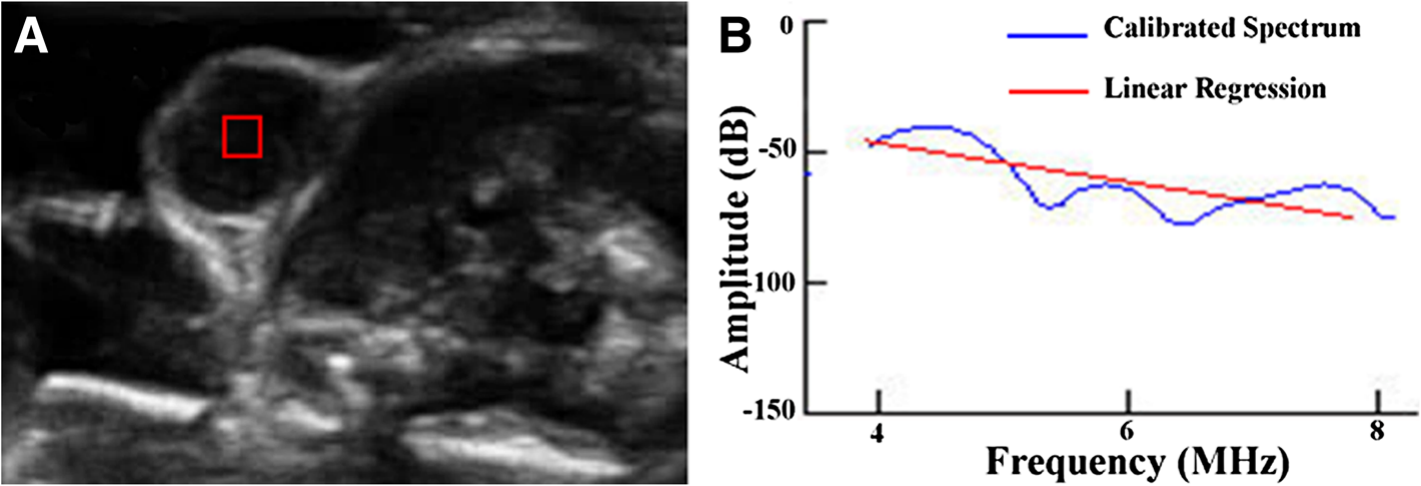
**Ultrasound image and power spectrum.** (**A**) Ultrasound image of the MCF7 tumor with the ROI located in the subcutaneous tumor tissue and (**B**) the corresponding calibrated power spectrum (blue curve) with linear regression (red line).

The power spectrum was obtained by averaging the results from the independent scan lines. This power spectrum was divided by the power spectrum of the echo from a calibration target. A quartz flat was used as the calibration target and the perpendicular reflection off the quartz flat located at the focal point of the transducer was used to derive the power spectrum. Linear regression analysis was applied to the calibrated spectral amplitude to provide a best-fit line (Figure 1) [19]. To assess the role of ultrasonic spectrum analysis in monitoring tumor response to chemotherapy, two spectral parameters from the regression analysis were computed: the spectral slope (SS) and the midband fit (MBF). The SS is the slope of the linear regression of the calibrated spectrogram and the MBF is the value of the regression fit at the center frequency over which the spectrum was measured. The spectral parameters can potentially be mathematically related to the physical characteristics of ultrasound scatterers, such as the size and concentration of the ultrasound scatterers [20]. Further details on the theoretical and signal analysis considerations and the relationship between spectral parameters and tissue microstructure can be found elsewhere [19,21].

### Histopathologic examination

At the end of the experiment, mice were sacrificed using the standard method. Tumors were removed and fixed in 10% buffered formalin before paraffin processing. The tumor specimens were sectioned (5 μm) at the largest cross sections corresponding to the ultrasound imaging planes. Sections were stained with hemotoxylin and eosin (H&E) and assessed microscopically for changes in cell morphology.

**Figure 2:**
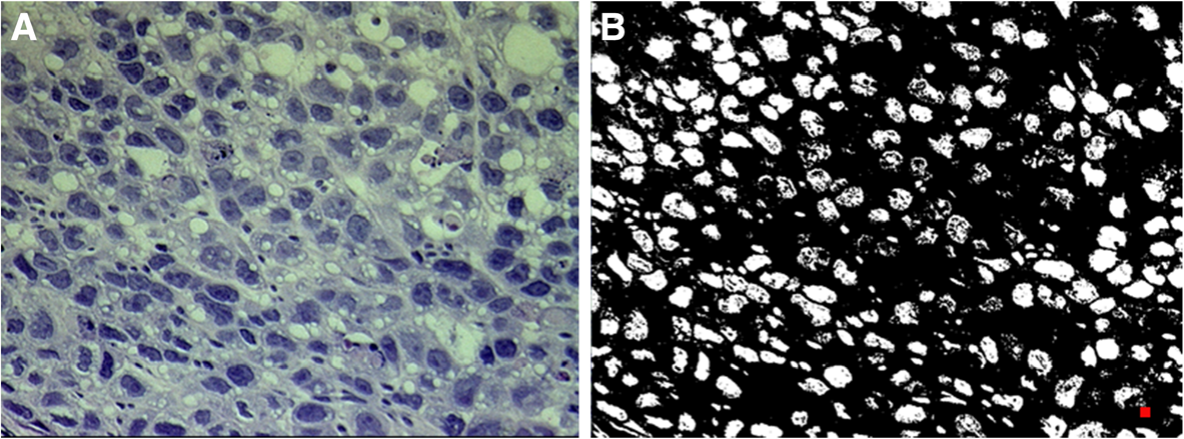
**Histologic section and binary image.** (**A**) A hemotoxylin and eosin (H&E) stained image of the MCF-7 tumor and (**B**) the corresponding binary image indicating the presence of nuclei were identified using Image Pro Plus. The red square showed the area threshold used for counting the nuclei.

Regions with the highest density of tumor cell nuclei were located by scanning the tissue sections under a 40x power microscope. After identification of the regions of highest density, ten different fields were randomly chosen within these regions at 400x power. The 400x histology images were then analyzed to measure the density of tumor cell nuclei by counting the number of nuclei in each image using Image Pro Plus software (Image Pro Plus 6.0, Media Cybernetics, Silver Spring, MD, USA) (Figure 2). The H&E stained image segmentation was based on the following Hue-Saturation-Intensity (HSI) parameters: Hue (0–255), Saturation (0–255) and Intensity (0–120). The segmented areas in the images were filtered to count blue nuclei. This filtering used thresholds as follows: area (minimum = 50 pixels) and box x/y (minimum = 0.5; maximum =2). The “split objects” function was used to separate cells touching each other. The average count of ten 400x images was used for the statistical analysis.

### Statistical analysis

All statistical analyses were performed using SPSS version 16.0 (SPSS, Inc, Chicago, IL, USA). The Kolmogorov-Smirnov test was applied for evaluation of normal distribution and the Levene test for evaluation of homogeneity of variance. Student’s *t*-test was used to determine the significant differences in measurement data between the treated and control tumors. A leave-one-out cross-validation method was used to test the ability of slope and midband-fit to distinguish treated tumors from control tumors. A *p* value of 0.05 or less was considered as being statistically significant.

## Results

### Effect of adriamycin on tumor growth

After treatment for 7 days, the mean tumor volumes of the treated and control tumors were 0.08 ± 0.03 cm^3^ and 0.17 ± 0.08 cm^3^, respectively. Treatment with adriamycin (4 mg/kg once daily) significantly reduced tumor growth in comparison to the control group (*p* =0.003).

### Changes in ultrasonic spectral parameters

**Figure 3:**
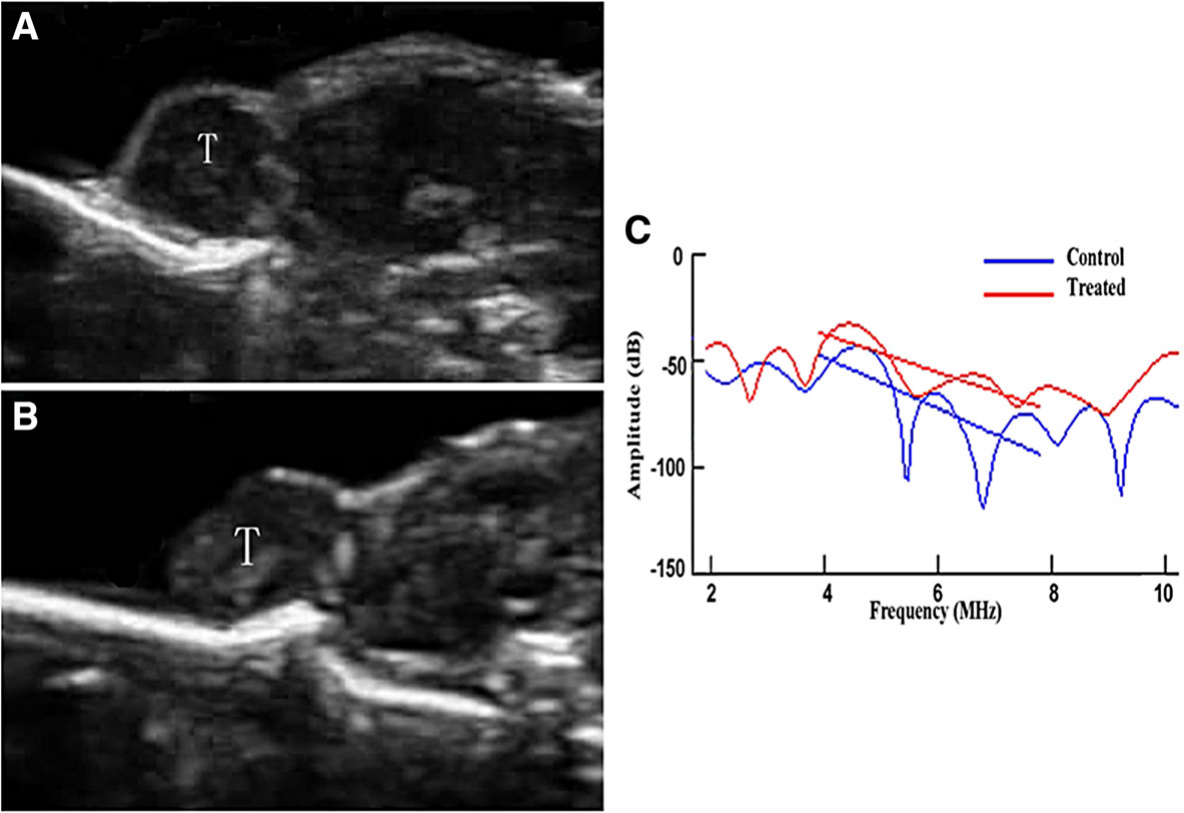
**Representative ultrasound images and corresponding ultrasound spectral parameters.** Ultrasound images for (**A**) a control tumor and (**B**) a treated tumor, and the corresponding ultrasound spectral parameter (**C**) characterization of tumor responses to chemotherapy. The ultrasound images showed a noticeably brighter grayscale intensity in treated tumors (**B**) relative to control tumors (**A**). (**C**) Ultrasonic spectral analysis indicated a separation of the regression line between the control and the treated tumors.

After treatment for 7 days, the mean gray scale intensity of the control and the treated tumors in conventional B-mode ultrasound images was 15.40 ± 3.86 units and 19.00 ± 1.25 units, respectively. Treatment with adriamycin (4 mg/kg once daily) significantly increased the gray scale intensity of the conventional B-mode ultrasound images as compared with control tumors (*p* = 0.009). Spectrum analysis of frequency-dependent backscattered radiofrequency data showed the difference in spectral parameters between the treated and control tumors. The mean spectral slope of the control and treated tumors was −10.66 ± 2.96 dB/MHz and −5.49 ± 2.69 dB/MHz, respectively. Seven days treatment with adriamycin (4 mg/kg once daily) significantly increased spectral slope (by 48.5%) in comparison to the control group (*p* < 0.001). The mean MBF of the control and treated tumors was −57.10 ± 7.68 and −49.81 ± 5.40 dB, respectively. MBF was significantly increased (by 12.8%) after 7 days when the treatment tumor group was compared with the control group (*p* = 0.013) (Figure 3). In distinguishing between treated and control tumors using “leave-one-out” cross-validation, the correctly classified rate of slope and MBF was 87.5%, and the sensitivity and specificity were 83.3% and 91.7%, respectively.

### Histological changes

**Figure 4:**
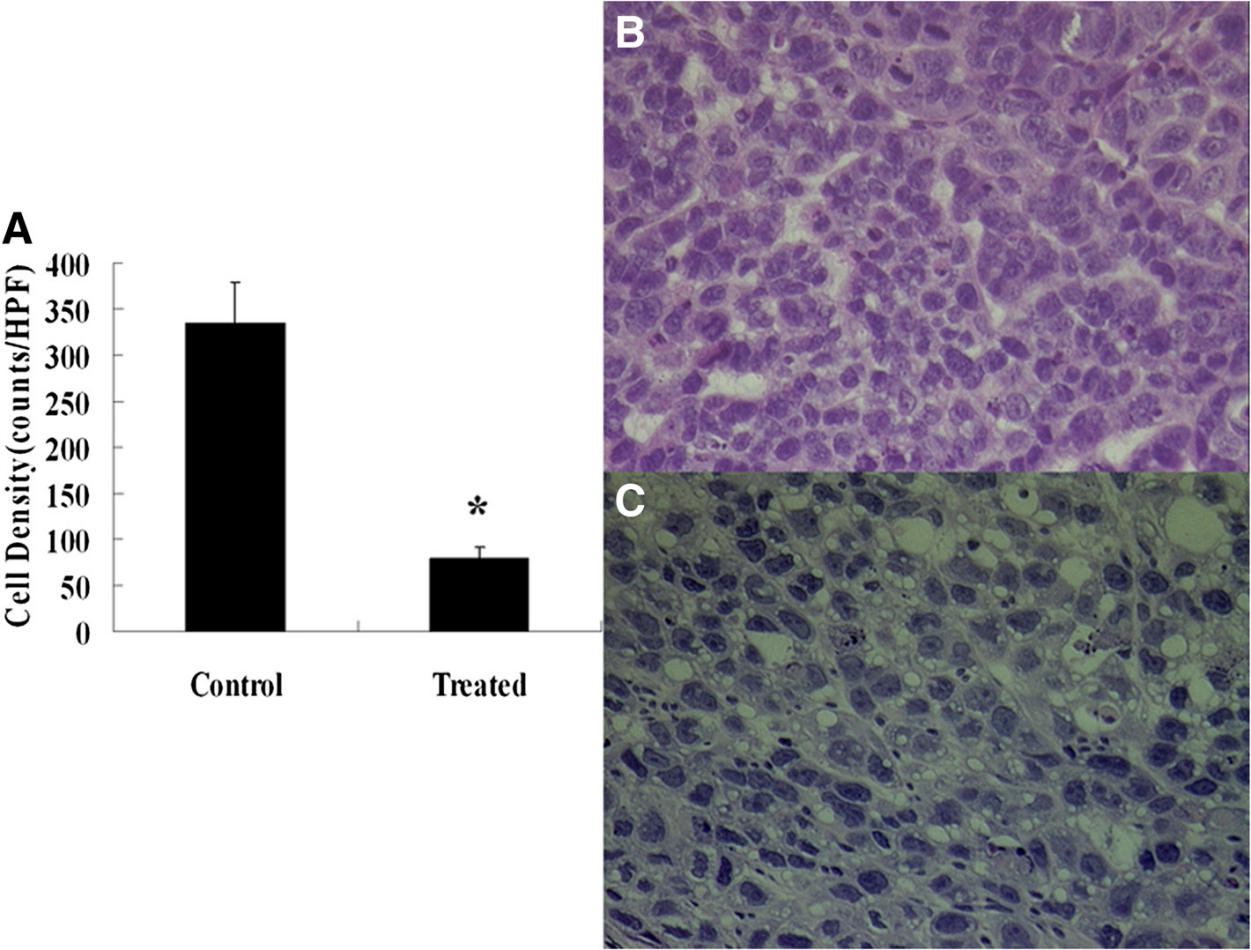
**Histopathologic analysis of tumor cell nuclei density.** (**A**) The graph displays the mean density of tumor cell nuclei in the control and treated tumors (* = *p* < .001). (**B**) and (**C**) Representative photomicrographs of hemotoxylin and eosin stained sections of the control (**B**) and treated (**C**) tumors. Staining revealed microstructural changes in the tumor treated with adriamycin, including a decreased density of cell nuclei, and cytoplasmic and nuclear vacuolation, and clumping of nuclear chromatin (original magnification, ×400).

**Figure 5:**
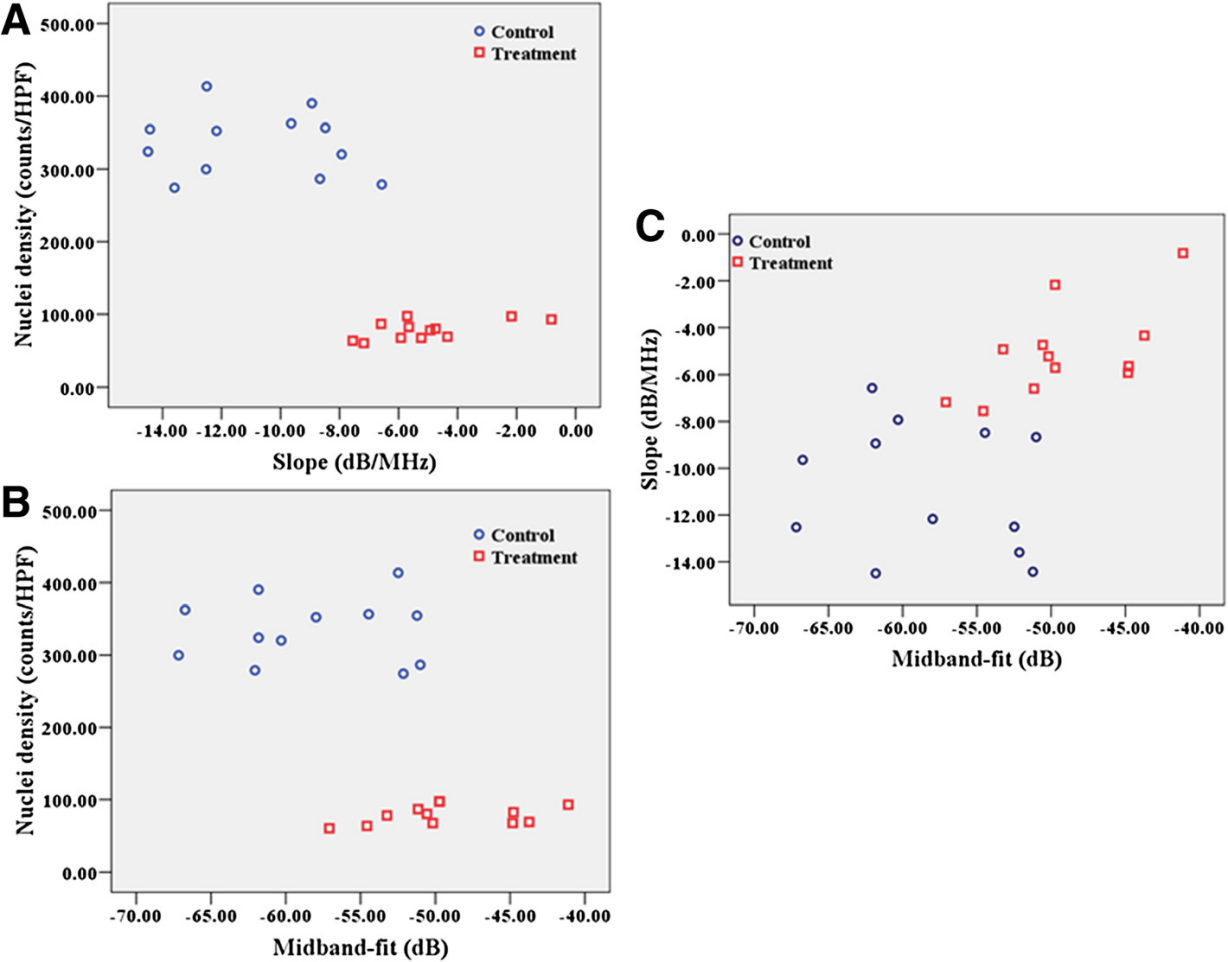
**Scatter plots of changes in spectral parameters.** (**A**) Spectral slope, (**B**) midband-fit versus cell nuclei density and (**C**) spectral slope versus midband-fit.

The most prominent histological changes after chemotherapy in the treated tumors were related to the density of tumor cell nuclei, nuclear size and the extent of cytoplasmic and nuclear vacuolation of the tumor cells. The number of tumor cell nuclei evaluated in histological slice from treated tumors was 78.51 ± 13.11 counts per high-power field (HPF) and in control tumors was 334.50 ± 44.57 counts per HPF. Treatment with adriamycin (4 mg/kg once daily) significantly reduced the density of tumor cell nuclei in comparison to the control group (*p* < 0.001). H&E staining revealed other microstructural change in treated tumors, involving nuclear structure manipulation (condensation and fragmentation) (Figures 4 and 5).

### Correlation between ultrasonic spectral parameters and histological results

The density of cell nuclei density was found to be negatively correlated with spectral slope (r = −0.670, *p* < 0.001) and MBF (r = −0.450, *p* = 0.027).

## Discussion

Reduction in tumor size is a late sign of effective chemotherapy, early response to chemotherapy is difficult to assess using conventional radiographic modalities [22]. Currently many cancer treatment regimens require several courses of chemotherapy before it can be determined whether or not the treatment has been effective. The availability of non-invasive methods for predicting and/or detecting therapeutic response to chemotherapy at an early stage of treatment would facilitate the rational design and individualization of therapy protocols for cancer patients and allow early transition to second-line therapy. The present study was undertaken to assess the usefulness of ultrasonic spectral analysis in the evaluation of conventional chemotherapy in a murine breast cancer model using tumor volume and the density of tumor cell nuclei as gold standards. This study has shown for the first time, using a clinically available 6 MHz ultrasound transducer, that chemotherapy effects can be characterized by means of ultrasonic spectral analysis in preclinical mouse cancer models. The changes in spectral parameters were interpreted as occurring as a direct consequence of cell death and changes in the density of cell nuclei after chemotherapy. This method was able to detect changes in the solid tumor microstructure after chemotherapy and, consequently has the potential to detect tumors that are responsive to treatment earlier than using conventional methods.

In the current study, histologic analysis revealed that the most prominent microstructural changes after chemotherapy were related to the density of cell nuclei, cytoplasmic and nuclear vacuolation and clumping of nuclear chromatin as indicated by the H&E staining; these changes are frequently seen in breast cancer following neoadjuvant chemotherapy [23,24]. Our histologic observations suggested that the main changes in the ultrasonic spectral parameters after chemotherapy were related to microstructural changes regarding both the density of cell nuclei and cytomorphologic changes.

It has long been suggested that ultrasound backscatter variables may relate to the physical properties of tissues [20]. Ultrasonic spectral analysis techniques have been used by many investigators to add information regarding tissue microstructure to images generated by conventional ultrasound [25]. The spectral slope is an indicator of effective scatterer shape and size and an increase in spectral slope corresponds to a decrease in effective scatterer size [26]. The observed changes in the spectral slope after chemotherapy could be suggestive of structural changes in the tumor cells after treatment, namely cell shrinkage, nuclear condensation and fragmentation. The MBF is another measure of ultrasound backscatter and depends on multiple factors, including scatterer shape, size, concentration, and the acoustic impedance change between the acoustic scatterer and the surrounding medium [26]. Increase in MBF was seen in treated tumors after chemotherapy relative to control tumors. The mechanism behind this increase was broadly linked to changes in cell and nuclear morphology observed histologically after cell death. The strength of ultrasound backscatter depends both on scatterer size and differences in the mechanical properties of the scatterer, the surroundings (compressibility and density) and the scatterer number density (e.g., how many scatterers there are per unit volume) [15]. The changes in cell and nuclear sizes during the sequence of cell death resulted in an increase in the variance of cell sizes; a previous study by Vlad et al. [27] demonstrated that an increase in cellular size variance contributes to the increase in ultrasound backscatter during cell death. The decrease in the density of tumor cell nuclei and extensive changes in cytoplasmic and nuclear vacuolation of the tumor cells might result in changes in acoustic impedance, contributing to the increase in MBF.

Ultrasonic parameters, including spectral slope and MBF, have previously been used to characterize diseased tissue or tissue and cell samples exposed to different therapeutic agents [14-17,27,28]. Kolios and colleagues used ultrasonic spectral analysis to measure changes in the spectral slope and MBF *in vitro* for cell samples exposed to chemotherapeutic drugs [29]. Significant increases in spectral slope and MBF in treated cells were observed after exposure to chemotherapy that were in close agreement with theoretical predictions. However, more complex histological changes were observed in *in vivo* in mouse breast cancer models after chemotherapy than in vitro in cell samples exposed to chemotherapeutic drugs. In our study, other than cell shrinkage and nuclear condensation and fragmentation, the most prominent microstructural changes observed after chemotherapy were related to the decrease in the density of tumor cell nuclei, and cytoplasmic and nuclear vacuolation as revealed using H&E staining. Moreover, high-frequency ultrasound has been used in previous studies to detect apoptosis *in vivo* in cell samples and animal systems exposed to different anticancer therapies [14-17,29]. A penetration depth of 2–5 cm at frequencies of 10–30 MHz limits the applicability of this technique to superficial regions. The present study confirmed that ultrasonic spectral analysis using a lower frequency of 6 MHz could be used to monitor changes in tumor microstructure in a mouse breast cancer tumor model after chemotherapy. Sadeghi-Naini and colleagues used ultrasonic spectral analysis with a Sonix RP system at an approximate center frequency of 7 MHz to examine neoadjuvant chemotherapeutic response in breast cancer patients, and their preliminary results were promising [18]. Lower frequency ultrasound penetrates much deeper, and previous studies have confirmed that the backscattered data from low frequency ultrasound could be potentially subjected to the same analysis to provide information regarding structural changes in the tissue at the cellular level [30-33].

Increased tumor cell death occurring early during the course of treatment, in both preclinical and clinical studies, has been shown to be a good prognostic indicator of outcome [5,6]. As a result of tumor cell death, the density of tumor cell nuclei will decrease significantly after chemotherapy [23,24,34]; this has become a marker for the detection of early indications of tumor response to chemotherapy [4]. Noninvasive diffusion-weighted MRI is a well studied imaging technique for quantifying the increase in the apparent diffusion coefficient of water caused by a decrease in tumor cell density within 2–4 days, prior to visible changes in tumor morphology or size in patients with breast [35], brain [36], and ovarian [8] cancers that responded to treatment. However, the cost of diffusion-weighted MRI limits its extensive use in monitoring tumor response to cancer therapies. In contrast, in the measurement of changes in water diffusion caused by cell death, ultrasonic spectral analysis provides a simple imaging approach that can directly characterize tumor microstructural changes (decreased density of tumor cell nuclei) after chemotherapy.

A potential limitation of our technique was that there were potential variations in matching the ultrasound image planes with the histological slices, due to differences in slice thickness between the ultrasound scanner and histological sections. In order to make ultrasound data correspond more closely to histologic measurement, the largest cross-section planes were used in both techniques. Another limitation was that there was no compensation for attenuation in our study, However, because of the fact that the tumors were close to the skin surface, and the center of the ROI was located at approximately 0.2 cm below the skin surface, attenuation compensation will not likely lead to significant changes in the results for a 6 MHz transducer. Moreover, the limiting scatterer size for a 6-MHz linear transducer will be about 85 μm (13), which is much larger than the nuclear diameter (about 15 μm); consequently, it was difficult to identify the histological texture of the scatterer for the 6-MHz transducer, and therefore to specify the histological changes that caused the changes in the spectral parameters.

## Conclusions

In conclusion, this study indicated that ultrasonic spectral analysis provided a simple way to characterize tumor microstructural changes after chemotherapy using a clinically available 6 MHz ultrasound transducer. A significant increase in spectral slope and MBF were detected using this noninvasive imaging technique after chemotherapy. This would allow tumor imaging before and at multiple times during treatment without the need for injecting specialized contrast agents as is required using other techniques (e.g. PET, dynamic contrast-enhanced CT and dynamic contrast-enhanced MRI). This noninvasive technique shows considerable potential in the early assessment of tumor response to chemotherapy.

## Competing interests

The authors declare that there is no conflict of interest that could influence the impartiality of the research reported.

## Authors’ contributions

JHZ conceived the study; CYL, JWW, LHC, WZ, YC, AHL and JHZ performed the experiments, CYL, JWW, LHC and JHZ contributed to data analysis; LHC and JHZ wrote the paper. All authors read and approved the final manuscript.

## Pre-publication history

The pre-publication history for this paper can be accessed here:

http://www.biomedcentral.com/1471-2407/13/302/prepub

## References

[B1] Groheux D Giacchetti S Espié M Rubello D Moretti JL Hindié E Early monitoring of response to neoadjuvant chemotherapy in breast cancer with 18F-FDG PET/CT: defining a clinical aim Eur J Nucl Med Mol Imaging 2011 38 419 425 10.1007/s00259-010-1660-5 21072510

[B2] Ah-See ML Makris A Taylor NJ Harrison M Richman PI Burcombe RJ Stirling JJ D'Arcy JA Collins DJ Pittam MR Ravichandran D Padhani AR Early changes in functional dynamic magnetic resonance imaging predict for pathologic response to neoadjuvant chemotherapy in primary breast cancer Clin Cancer Res 2008 14 6580 6589 10.1158/1078-0432.CCR-07-4310 18927299

[B3] Bellomi M Petralia G Sonzogni A Zampino MG Rocca A CT perfusion for the monitoring of neoadjuvant chemotherapy and radiation therapy in rectal carcinoma: initial experience Radiology 2007 244 486 493 10.1148/radiol.2442061189 17641369

[B4] Brindle K New approaches for imaging tumour responses to treatment Nat Rev Cancer 2008 8 94 107 18202697 10.1038/nrc2289

[B5] Ellis PA Smith IE McCarthy K Detre S Salter J Dowsett M Preoperative chemotherapy induces apoptosis in early breast cancer Lancet 1997 349 849 10.1016/S0140-6736(05)61752-7 9121265

[B6] Chang J Ormerod M Powles TJ Allred DC Ashley SE Dowsett M Apoptosis and proliferation as predictors of chemotherapy response in patients with breast carcinoma Cancer 2000 9 2145 2152 11147583

[B7] Hamstra DA Galbán CJ Meyer CR Johnson TD Sundgren PC Tsien C Lawrence TS Junck L Ross DJ Rehemtulla A Ross BD Chenevert TL Functional diffusion map as an early imaging biomarker for high-grade glioma: correlation with conventional radiologic response and overall survival J Clin Oncol 2008 26 3387 3394 10.1200/JCO.2007.15.2363 18541899 PMC3266717

[B8] Kyriazi S Collins DJ Messiou C Pennert K Davidson RL Giles SL Kaye SB Desouza NM Metastatic ovarian and primary peritoneal cancer: assessing chemotherapy response with diffusion-weighted MR imaging–value of histogram analysis of apparent diffusion coefficients Radiology 2011 261 182 192 10.1148/radiol.11110577 21828186

[B9] Liu T Mansukhani MM Benson MC Ennis R Yoshida E Schiff PB Zhang P Zhou J Kutcher GJ A feasibility study of novel ultrasonic tissue characterization for prostate-cancer diagnosis: 2D spectrum analysis of in vivo data with histology as gold standard Med Phys 2009 36 3504 3511 10.1118/1.3166360 19746784 PMC2832027

[B10] Liu T Lizzi FL Silverman RH Kutcher GJ Ultrasonic tissue characterization using 2-D spectrum analysis and its application in ocular tumor diagnosis Med Phys 2004 31 1032 1039 10.1118/1.1690196 15191289 PMC2838231

[B11] Yang M Krueger TM Miller JG Holland MR Characterization of anisotropic myocardial backscatter using spectral slope, intercept and midband fit parameters Ultrason Imaging 2007 29 122 134 10.1177/016173460702900204 17679326

[B12] Kumon RE Pollack MJ Faulx AL Olowe K Farooq FT Chen VK Zhou Y Wong RC Isenberg GA Sivak MV Chak A Deng CX In vivo characterization of pancreatic and lymph node tissue by using EUS spectrum analysis: a validation study Gastrointest Endosc 2010 71 53 63 10.1016/j.gie.2009.08.027 19922913 PMC2900783

[B13] Lizzi FL Ultrasonic scatterer-property images of the eye and prostate Proc 1997 IEEE Ultrasonics Symp 1997 1109 1116

[B14] Vlad RM Brand S Giles A Kolios MC Czarnota GJ Quantitative ultrasound characterization of responses to radiotherapy in cancer mouse models Clin Cancer Res 2009 15 2067 2075 10.1158/1078-0432.CCR-08-1970 19276277

[B15] Banihashemi B Vlad R Debeljevic B Giles A Kolios MC Czarnota GJ Ultrasound imaging of apoptosis in tumor response: novel preclinical monitoring of photodynamic therapy effects Cancer Res 2008 68 8590 8596 10.1158/0008-5472.CAN-08-0006 18922935

[B16] Lee J Karshafian R Papanicolau N Giles A Kolios MC Czarnota GJ Quantitative ultrasound for the monitoring of novel microbubble and ultrasound radiosensitization Ultrasound Med Bio 2012 38 1212 1221 10.1016/j.ultrasmedbio.2012.01.028 22579547

[B17] Hwang JY Park J Kang BJ Lubow DJ Chu D Farkas DL Shung KK Medina-Kauwe LK Multimodality imaging in vivo for preclinical assessment of tumor-targeted doxorubicin nanoparticles PLoS One 2012 7 e34463 10.1371/journal.pone.0034463 22509306 PMC3317981

[B18] Sadeghi-Naini A Falou O Czarnota GJ Quantitative ultrasound spectral parametric maps: Early surrogates of cancer treatment response Prof. 34th Annual Intl. Conf. Proc. IEEE Eng Med Biol Soc 2012 2672 2675 10.1109/EMBC.2012.6346514 23366475

[B19] Lizzi FL Greenebaum M Feleppa EJ Elbaum M Coleman DJ Theoretical framework for spectrum analysis in ultrasonic tissue characterization J Acoust Soc Am 1983 73 1366 1373 10.1121/1.389241 6853848

[B20] Lizzi FL Astor M Feleppa EJ Shao M Kalisz A Statistical framework for ultrasonic spectral parameter imaging Ultrasound Med Biol 1997 23 1371 1382 10.1016/S0301-5629(97)00200-7 9428136

[B21] Lizzi FL Astor M Liu T Deng C Coleman DJ Silverman RH Ultrasonic spectrum analysis for tissue assays and therapy evaluation Int J Imaging Syst Technol 1997 8 3 10 10.1002/(SICI)1098-1098(1997)8:1<3::AID-IMA2>3.0.CO;2-E

[B22] Hlatky L Hahnfeldt P Folkman J Clinical application of antiangiogenic therapy: microvessel density, what it does and doesn’t tell us J Natl Cancer Inst 2002 94 883 893 10.1093/jnci/94.12.883 12072542

[B23] Rajan R Esteva FJ Symmans WF Pathologic changes in breast cancer following neoadjuvant chemotherapy: implications for the assessment of response Clin Breast Cancer 2004 5 235 238 10.3816/CBC.2004.n.028 15335458

[B24] Rajan R Poniecka A Smith TL Yang Y Frye D Pusztai L Fiterman DJ Gal-Gombos E Whitman G Rouzier R Green M Kuerer H Buzdar AU Hortobagyi GN Symmans WF Change in tumor cellularity of breast carcinoma after neoadjuvant chemotherapy as a variable in the pathologic assessment of response Cancer 2004 100 1365 1373 10.1002/cncr.20134 15042669

[B25] Czarnota GJ Kolios MC Hunt JW Sherar MD Ultrasound imaging of apoptosis. DNA-damage effects visualized Methods Mol Biol 2002 203 257 277 12073448 10.1385/1-59259-179-5:257

[B26] Lizzi FL King DL Rorke MC Hui J Ostromogilsky M Yaremko MM Feleppa EJ Wai P Comparison of theoretical scattering results and ultrasonic data from clinical liver examinations Ultrasound Med Biol 1988 14 377 385 10.1016/0301-5629(88)90073-7 3051612

[B27] Vlad RM Saha RK Alajez NM Ranieri S Czarnota GJ Kolios MC An increase in cellular size variance contributes to the increase in ultrasound backscatter during cell death Ultrasound Med Biol 2010 36 1546 1558 10.1016/j.ultrasmedbio.2010.05.025 20800181

[B28] Taggart LR Baddour RE Giles A Czarnota GJ Kolios MC Ultrasonic characterization of whole cells and isolated nuclei Ultrasound Med Biol 2007 33 389 401 10.1016/j.ultrasmedbio.2006.07.037 17257739

[B29] Kolios MC Czarnota GJ Lee M Hunt JW Sherar MD Ultrasonic spectral parameter characterization of apoptosis Ultrasound Med Biol 2002 28 589 597 10.1016/S0301-5629(02)00492-1 12079696

[B30] Oelze ML Zachary JF O'Brien WD Jr Parametric imaging of rat mammary tumors in vivo for the purposes of tissue characterization J Ultrasound Med 2002 21 1201 1210 12418761 10.7863/jum.2002.21.11.1201

[B31] Czarnota GJ Papanicolau N Lee J Karshafian R Giles A Kolios MC **Novel low-frequency ultrasound detection of apoptosis in vitro and in vivo** [abstract] Ultrason Imaging 2008 29 237 238

[B32] Zhou J Zhang P Osterman KS Woodhouse SA Schiff PB Yoshida EJ Lu ZF Pile-Spellman ER Kutcher GJ Liu T Implementation and validation of an ultrasonic tissue characterization technique for quantitative assessment of normal-tissue toxicity in radiation therapy Med Phys 2009 36 1643 1650 10.1118/1.3103935 19544781 PMC2736706

[B33] Papanicolau N Karshafian R Sadeghian A Kolios M Czarnota G Conventional frequency evaluation of tumor cell death in response to treatment in vivo (abstract) J Acoust Soc Am 2010 128 2365

[B34] Symmans WF Peintinger F Hatzis C Rajan R Kuerer H Valero V Assad L Poniecka A Hennessy B Green M Buzdar AU Singletary SE Hortobagyi GN Pusztai L Measurement of residual breast cancer burden to predict survival after neoadjuvant chemotherapy J Clin Oncol 2007 25 4414 4422 10.1200/JCO.2007.10.6823 17785706

[B35] Park SH Moon WK Cho N Song IC Chang JM Park IA Han W Noh DY Diffusion-weighted MR imaging: pretreatment prediction of response to neoadjuvant chemotherapy in patients with breast cancer Radiology 2010 257 56 63 10.1148/radiol.10092021 20851939

[B36] Chenevert TL Stegman LD Taylor JM Robertson PL Greenberg HS Rehemtulla A Ross BD Diffusion magnetic resonance imaging: an early surrogate marker of therapeutic efficacy in brain tumors J Natl Cancer Inst 2000 92 2029 2036 10.1093/jnci/92.24.2029 11121466

